# Prurigo Nodularis after Radiotherapy: An Isoradiotopic Response?

**DOI:** 10.1155/2018/9186745

**Published:** 2018-08-13

**Authors:** Caspar Weel Krammer, Rami Mossad Ibrahim

**Affiliations:** ^1^Department of Plastic Surgery, Hospital South West Jutland, Finsensgade 35, 6700 Esbjerg, Denmark; ^2^Department of Plastic Surgery, Herlev Hospital, Herlev Ringvej 75, 2730 Herlev, Denmark

## Abstract

Prurigo nodularis is a rare and chronic skin disorder with multiple, pruritic, and firm nodules. The exact pathophysiology is still unknown. Skin disorders appearing at sites of radiation can be defined as isoradiotopic. A 35-year-old male had developed a skin lesion in the left submandibular area on a base of irradiated skin which was initially suspected as a skin malignancy. The patient had a history of undifferentiated nasopharyngeal cancer with lymph node involvement which was treated by radiochemotherapy thirteen years previously. Histological examination confirmed that it was a case of prurigo nodularis which subsequently evolved at distant sites. This presentation may suggest a case of an isoradiotopic response.

## 1. Introduction

Prurigo nodularis (PN) first described in 1907 by Hyde, is a chronic skin condition characterized by multiple, symmetric, pruritic, and firm nodules in the skin [1]. Irradiated areas are believed to be more prone to the onset of secondary dermatoses, known as an isoradiotopic response [2]. In this report, we describe an atypical presentation of PN in a 35-year-old male patient with a history of radiochemotherapy for nasopharyngeal cancer with lymph node involvement. Thirteen years after radiotherapy he developed localized PN at the site of radiotherapy and subsequent distant multiple, pruritic nodules.

## 2. Case Report

**Figure 1:**
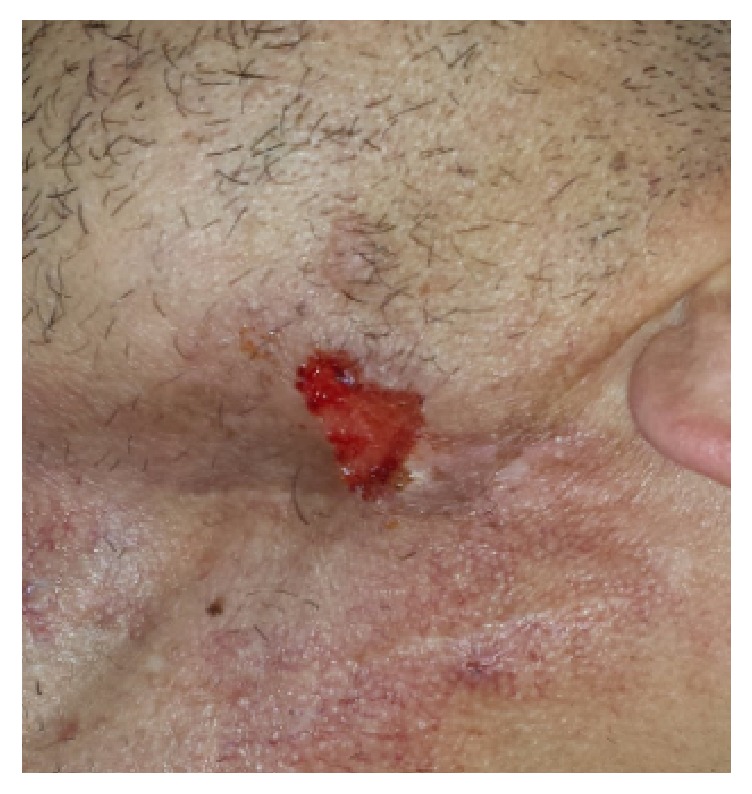
Prurigo nodularis. A 20x10mm lesion at the site of previous radiation therapy near the mandible.

A 35-year-old man was referred to plastic surgical department with a progressive, ulcerating lesion near the angle of the mandible suspicious for skin carcinoma (Figure 1). It had gradually grown to 20 mm in diameter over four months. Thirteen years previously, the patient was diagnosed with an undifferentiated nasopharyngeal cancer with lymph node involvement classified as T2aN2M0. The patient was treated with radiochemotherapy and had no recurrence. The radiation therapy had led to osteonecrosis and chronic radiation-induced dermatitis/fibrosis of the skin at the site of the radiotherapy. After the primary lesion emerged the patient developed multiple 5-6mm tumors on the extensor side of arms, lower limbs, and postauricular, which clinically presented as prurigo nodularis. The patient did not have a personal or family history of skin disorders.

**Figure 2:**
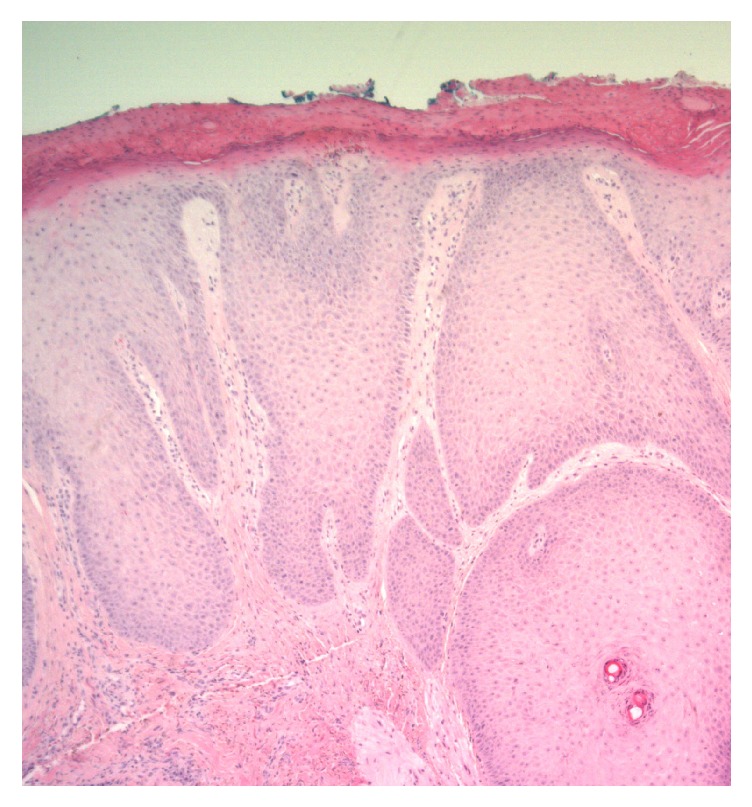
Prurigo nodularis. Hyperkeratotic and parakeratotic epidermis.

A pouch biopsy was taken from the lesion near the mandible in order to rule out radiotherapy induced malignancy. Subsequent histologic examination identified it as prurigo nodularis (Figure 2). The pathological examination showed a hyperkeratosis and parakeratosis epidermis with irregular acanthosis. The patient was offered a referral to a dermatologist for evaluation but expressed a desire for surgical treatment. The lesion was excised with a close margin in local anesthesia and the defect was closed directly. This was once again histologically confirmed as prurigo nodularis. The patient healed without any complications. The remaining nodules on the limbs and postauricular were referred to a dermatologist.

## 3. Discussion

Roucco et al. used the term immunocompromised district (ICD) to describe a skin area with local dysregulation of the immune system, which can lead to vulnerability to different skin disorders [3]. An example of such vulnerability can be seen at the site of a previous dermatosis, usually postherpetic. This was described by Wolf et al. as an isotopic response where a new skin disorder appears at the site of a previous dermatosis [4]. A similar response for irradiated skin areas was termed as an isoradiotopic response by Shurman et al. [2]. By this terminology, secondary dermatoses appearing in fields of radiation treatment can be classified as an isoradiotopic response.

Irradiated skin can be linked to altered lymph flow, dysfunctional neuroimmune signaling due to reduction in peptidergic nerve fibers. These changes can interfere with the local immune responses of the irradiated skin, whereby the area becomes an ICD [5].

The concept of dermatoses evolving at sites of previous radiotherapy is not new. Irradiated area is known to be more prone to skin disorders, which includes secondary malignancies [6]. Lichen planus, bullous pemphigoid, and pemphigus have also been reported in irradiated areas [2]. Prurigo nodularis has been reported as an isotopic response in a healed herpes zoster scar; however, PN has not to our knowledge previously been seen as an isoradiotopic response [7].

The exact pathophysiology of PN is still unknown; however, it is believed that continuing scratching may trigger PN. Different causes of pruritus have been descripted in patients with PN and may both be focal (e.g., insect bites, folliculitis, and eczema), systemic (e.g., chronic kidney failure), neurological, or psychogenic [8, 9]. In particular, inflammatory dermatoses such as atopic dermatitis have been linked to PN [10]. All these causes are known to cause pruritus; however, the patient denied any symptoms (e.g., itching) in the irradiated area.

In this case the lesion was treated surgically, because it primary presented as a small lesion suitable for surgical treatment. Although many different treatments have been proposed with varying efficiency, surgical excision of PN is generally not considered as an option [1]. This is due to the nature of the disease with multiple nodules which would require extensive surgery. In this case the primary lesion was excised with success and minimal scarring. The patient did not experience local recurrence at 3 months of follow up, thereby making surgery a feasible option under certain circumstances.

PN have been reported in all ages; however, the elder population is mostly affected [10]. PN usually appears on the extensor areas of the limbs and it is seldom seen in the facial area [8]. Dermatological or systemic factors are usually present [9]. In the current report, a 35-year-old man developed a single lesion identified as PN in the left submandibular area on a base of irradiated skin, later PN evolved at distant sites. This could either suggest an isoradiotopic response or that PN was triggered by scratching, which the patient denied. This case may contribute to the belief that there is a distinctive phenomenon (i.e., isoradiotopic response) related to irradiated skin where secondary dermatoses can occur.
